# Restoring a butterfly hot spot by large ungulates refaunation: the case of the Milovice military training range, Czech Republic

**DOI:** 10.1186/s12862-021-01804-x

**Published:** 2021-04-30

**Authors:** Martin Konvička, David Ričl, Veronika Vodičková, Jiří Beneš, Miloslav Jirků

**Affiliations:** 1 Faculty of Sciences, University South Bohemia, Branišovská 31, 37005 České Budějovice, Czech Republic; 2 Biology Centre, Czech Academy of Sciences, Institute of Entomology, Branišovská 31, 37005 České Budějovice, Czech Republic; 3 Jílové u Prahy, Czech Republic; 4 European Wildlife, Šultysova 170, 28401 Kutná Hora, Czech Republic; 5 Česká Krajina O.P.S., Šultysova 170, 28401 Kutná Hora, Czech Republic

**Keywords:** *Bison bonasus*, *Bos taurus*, Climatic niche traits, *Equus caballus*, Lepidoptera conservation, Life history traits, Temperate grassland, Trophic rewilding

## Abstract

**Background:**

Refaunation/rewilding by large ungulates represents a cost-efficient approach to managing natural biotopes and may be particularly useful for areas whose biodiversity depends on disturbance dynamics and is imperilled by successional changes. To study impacts of refaunation on invertebrates, we focused on butterflies inhabiting the former military training range Milovice, Czech Republic, refaunated since 2015 by a combination of Exmoor pony (“wild” horse), Tauros cattle (“aurochs”), and European wisent.

**Methods:**

We analysed butterfly presence-absence patterns immediately after the military use termination (early 1990s), prior to the refaunation (2009), and after it (2016–19); and current abundance data gained by monitoring butterflies at refaunated and neglected plots. We used correspondence analysis for the presence-absence comparison and canonical correspondence analysis for the current monitoring, and related results of both ordination methods to the life history and climatic traits, and conservation-related attributes, of recorded butterflies.

**Results:**

Following the termination of military use, several poorly mobile species inclining towards oceanic climates were lost. Newly gained are mobile species preferring warmer continental conditions. The refaunated plots hosted higher butterfly species richness and abundances. Larger-bodied butterflies developing on coarse grasses and shrubs inclined towards neglected plots, whereas refaunated plots supported smaller species developing on small forbs.

**Conclusion:**

The changes in species composition following the cessation of military use were attributable to successional change, coupled with changes in species pool operating at larger scales. By blocking succession, large ungulates support butterflies depending on competitively poor plants. Restoring large ungulates populations represents a great hope for conserving specialised insects, provided that settings of the projects, and locally adapted ungulate densities, do not deplete resources for species with often contrasting requirements.

**Supplementary Information:**

The online version contains supplementary material available at 10.1186/s12862-021-01804-x.

## Background

In most continents, late-Pleistocene and early Holocene human pressure extirpated, or drastically reduced, the populations of large ungulate herbivores, which reshaped the ecological dynamics of entire biomes [38, 55]. This affected nutrient cycling [e.g., 109], fire regimes [e.g., 37,67], seed dispersal and germination [29, 102], and overall vegetation physiognomy [34, 109]. Subsequent activities of preindustrial agriculturalists and pastoralists resumed the role of large wild ungulates, maintaining the disturbance-succession dynamics exploited by numerous species, including invertebrates. Many enigmas and paradoxes encountered in European insects conservation—such as the affiliation of many taxa to purportedly “cultural” grasslands [90, 101], ancient ways of forests use [28, 105], frequently disturbed habitats [74, 95], or finely-grained landscapes [82, 83]—are resolved, once the large ungulates activity is factored in. The current biodiversity has evolved in a megafaunal world [6, 94]. Modern ecosystems are functionally incomplete, with entire trophic levels impoverished or missing, and if not actively managed, they fail to provide habitats for a sizeable portion of associated biota [27, 80].

The current refaunation /rewilding /naturalistic grazing movements [24, 40, 63, 81, 88] strive to reverse the transformation of ecosystems that started in the late Pleistocene and culminated with recent land use intensification [27, 49, 53, 70]. Although insect conservationists have long advocated habitat management by ungulate grazing [e.g., 23,68,84], relatively few megafauna refaunation projects have systematically targeted or monitored the impacts on insects [98]. Each refaunation project develops within specific sociocultural constraints, rarely allowing for proper replications [cf. 77; but see 40]. There is an urgent need to study refaunation effects on invertebrates, both as encouragement for others and as feedback for the wider conservation community [51, 77]. The effects may differ from targeted conservation grazing, a well-established practice for managing habitats of some insect species [e.g., 92]. Conservation grazing tends to be practiced on smaller scales, covering restricted seasonal time windows and under constant supervision of managers [14, 101], whereas refaunation operates on larger scales with minimum interventions.

**Fig. 1:**
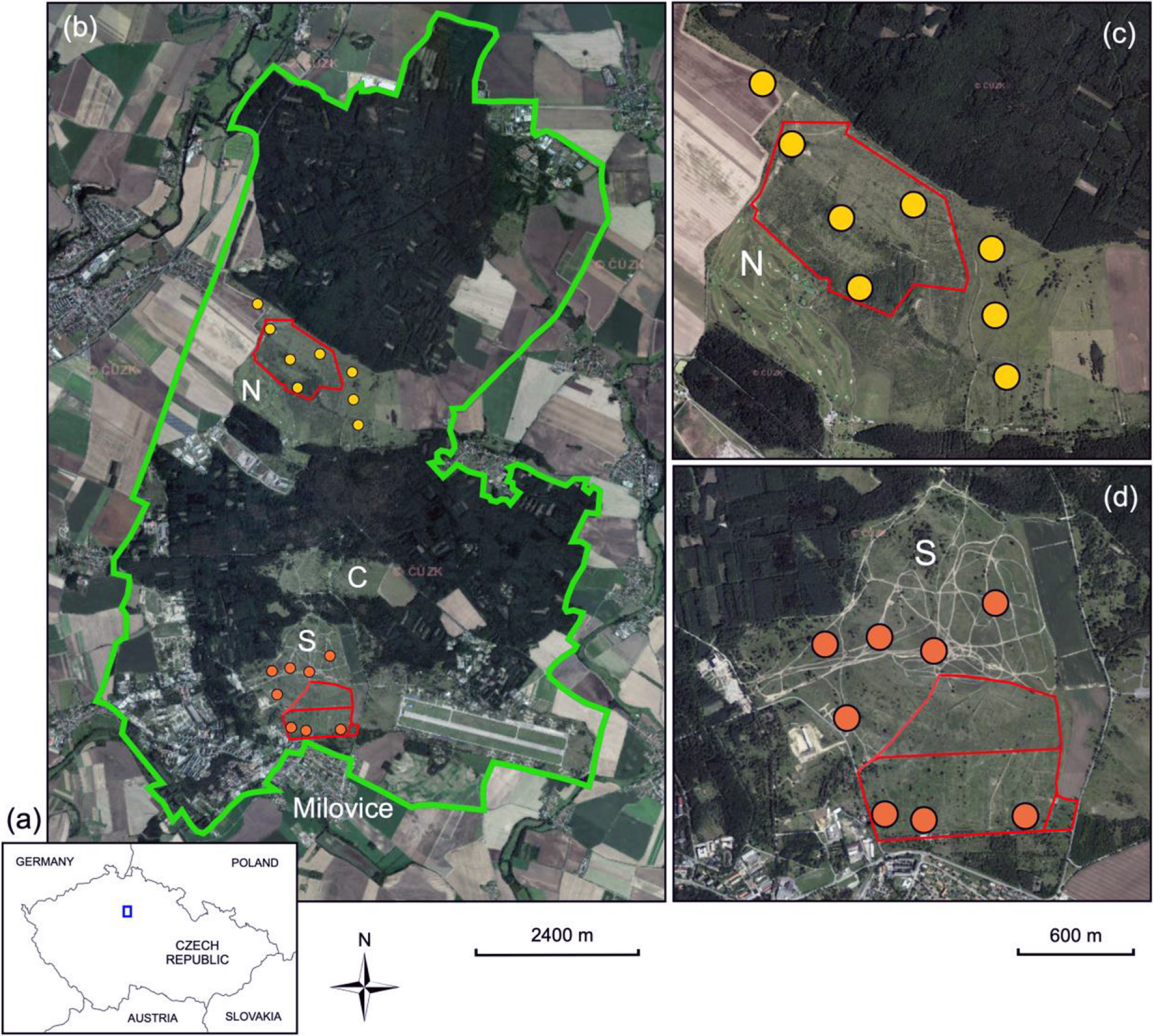
Former Milovice military training range. **a** The position of the range within the Czech Republic. **b** Aerial view of the wider area with the military range borders (green line), positions of the grasslands N, C, S, and borders of the two grazing reserves (red lines). **c**, **d** More detailed view at the 16 monitored plots within grasslands S and N. The three plots grazed by cattle for two years form the vertical line in eastern part of N. The background aerial photo is from mapy.seznam.cz, © Seznam.cz, a.s. Used according to general license agreement

The initial refaunation plans for the Czech Republic [53] aimed at large protected areas or actively used military training ranges. It was believed that in these large and biotically rich areas, restoring populations of large ungulates would be most feasible. The first refaunation project, however, has materialised on the relatively small scale of two grazing reserves within a disused military training range, in otherwise densely populated Central Bohemia (Fig. 1). Since 2015, three once near-extirpated components of native European megafauna are roaming on grasslands formerly used for army training: the European bison or wisent (*Bison bonasus*), a species rescued from near-certain extinction [72]; the back-bred “aurochs” in its restored Tauros form, derived from several taurine breeds of domestic cattle (*Bos taurus*) [41]; and the horse (*Equus caballus*), in an ancient feral Exmoor pony breed [3, 50].

Coincidently, the area was surveyed for butterflies immediately after the cessation of its military use [62], and again in the following decade, in a survey of abandoned military ranges [23]. The setting thus offers a unique opportunity to study butterfly assemblages’ responses to the abandonment of a military-used landscape, and to monitor effects of large ungulates refaunation on such assemblages. Such past-present comparison should also account for factors beyond locally operating disturbance-succession dynamics. An obvious candidate is changing climate, currently restructuring insect faunas on the continental scale [33, 106]. The mean temperatures increased in Cetral Europe by ≈1 °C during last two decades, although warming may not necessarily improve conditions for thermally demanding species, if microclimates cool down due to eutrophication [104]. Non-climatic drivers of species' distribution, such as large-scale land use changes causing population declines or increases [1], may also play a role.

A promising approach to generalisation from single-site results focuses on life history (= functional) traits of constituent species. It links habitat properties and the species composition of assemblages via species-specific traits [16, 59]. By linking species traits to results of habitat manipulation, it may disclose the mechanisms of species responses to habitat change [39, 84].

Here, we first compare butterfly records, life history traits, climatic niche traits, and conservation-related attributes from the three subsequent surveys: shortly after the military use termination, shortly before the refaunation by large ungulates, and under the large ungulates’ impact. For this past-present comparison, we hypothesised that cessation of military use was followed by losses of specialists of early-successional disturbed grounds, due to diminished ground disturbance by heavy vehicles (H1). Species gains should reflect the recent larger scale changes: Species with warmer climatic niches (H2) and/or species currently increasing their regional distribution extents (H3), should newly colonise the area. We then analyse results of the current monitoring of butterfly assemblages on refaunated versus neglected plots. We expected that by diversifying vegetation and creating more diverse disturbance regime, the whole-year grazing by wild ungulates should increase butterfly species richness and abundances compared to neglected plots (H4). The assemblage should shift from species associated with competitively dominant host plants, favoured by post-abandonment succession and typical for little disturbed vegetation, towards species associated with competitively inferior host plants and frequent disturbance by wild ungulates action (H5). We believe that this study is innovative in jointly considering the effects of site history, current species distribution drivers acting at larger scale, and large ungulates acting at small scale, on the composition of butterfly assemblages, and by utilising life history traits for gaining mechanistic understanding of the observed patterns.

## Results

### Past-present comparison

The early 1990s survey of the entire military range [62] detected 72 butterfly species (14 currently Red-listed); the interim survey of the sites N, C, S [23] detected 51 (6 Red-listed) species and the 2016–19 monitoring at the sites N and S detected 58 (7 Red-listed) species (Table 1, Additional file 1). The numbers are comparable only with caution. The 1990s earliest survey covered all biotopes in the area, including wooded parts outside the grasslands. It recorded a higher representation of arboreal species (n = 9) than the two latter surveys (4 and 5). The two latter surveys focused on grasslands, but while the interim survey consisted of five visits in a single year, the current monitoring consisted of 20 visits in four years. Still, even after exclusion of arboreal species and migrants whose abundances vary greatly among years, the earliest survey detected more species than the latter two surveys pooled.

**Table 1:** List of butterfly species (nomenclature and system: [107] recorded from the former Milovice military training range during the three consecutive surveys, split into respective localities if possible, with their Czech Republic Red-list [RL] status

Species	Abbreviation	RL status	Early 1990s	2009				2016–19		
			Entire area	Site S	Site C	Site N	Pooled	Site S	Site N	Pooled
Hesperidae										
*Carcharodus alceae*	*Calc*	NT	+	–	–	–	–	+	+	+
*Erynnis tages*	*Etag*	–	+	+	+	+	+	+	+	+
*Carterocephalus palaemon*	*Cpal*	–	+	+	+	+	+	+	+	+
*Pyrgus malvae*	*Pmal*	–	+	+	+	+	+	+	+	+
*Pyrgus armoricanus*		EN	+	–	–	–	–	–	–	–
*Ochlodes sylvanus*	*Osyl*	–	+	+	+	+	+	+	+	+
*Thymelicus lineola*	*Tlin*	–	+	+	+	+	+	+	+	+
*Thymelicus sylvestris*	*Tsyl*	–	+	+	+	+	+	+	+	+
*Thymelicus acteon*		EN	+	–	–	–	–	–	–	–
*Spialia sertorius*	*Sser*	VU	+	+	+	+	+	+	+	+
*Hesperia comma*	*Hcom*	VU	+	–	–	–	–	+	+	+
*Papilionidae*										
* Papilio machaon*	*Pmac*	–	+	+	+	+	+	+	+	+
* Iphiclides podalirius*	*Ipod*	NT	–	–	–	–	–	+	+	+
Pieridae		–								
*Pieris brassicae*	*Pbra*	–	+	+	+	+	+	+	+	+
*Pieris napi*	*Pnap*	–	+	+	+	+	+	+	+	+
*Pieris rapae*	*Prap*	–	+	+	+	+	+	+	+	+
*Leptidea juvernica*	*Ljuv*	–	+	+	+	+	+	+	+	+
*Gonepteryx rhamni*	*Grha*	–	+	+	+	+	+	+	+	+
*Colias alfacariensis*	*Calf*	VU	+	+	+	+	+	+	+	+
*Colias hyale*^$$^	*Chya*	–		–	–	–	–			
*Colias crocea*^$$^	*Ccro*	–	+	–	–	–	–	+	+	+
*Anthocharis cardamines*	*Acar*	–	+	+	+	+	+	+	+	+
*Pontia edusa*^$$^	*Pedu*	–	+	+	+	+	+	+	+	+
Nymphalidae										
*Apatura iris*^$^	*Airi*	–	+	–	–	–	–	+	–	+
*Apatura ilia*^$^		–	+	+	–	–	–	–	–	–
*Aglais urticae*	*Aurt*	–	+	+	+	+	+	+	+	+
*Nymphalis antiopa*^$^		–	+	+	+	+	+	–	–	–
*Nymphalis polychloros*^$^		–	+	–	–	–	–	–	–	–
*Vanessa atalanta*^$$^	*Vata*	–	+	+	+	+	+	+	+	+
*Vanessa cardui*^$$^	*Vcar*	–	+	+	+	+	+	+	+	+
*Inachis io*	*Iio*	–	+	+	+	+	+	+	+	+
*Araschnia levana*	*Alev*	–	+	+	+	+	+	+	+	+
*Polygonia c-album*	*Pc-a*	–	+	+	+	+	+	+	+	+
*Malitaea athalia*		NT	+	–	+	+	+	–	–	–
*Malitaea cinxia*		VU	+	–	–	–	–	–	–	–
*Issoria lathonia*	*Ilat*	–	+	+	+	+	+	+	+	+
*Argynnis aglaja*	*Aagl*	–	+	+	+	+	+	–	+	+
*Argynnis adippe*	*Aadi*	–	+	+	–	+	–	+	–	+
*Argynnis paphia*	*Apap*	–	+	+	+	+	+	+	+	+
*Boloria dia*	*Bdia*	–	+	+	+	+	+	+	+	+
*Boloria selene*		NT	+	–	–	–	–	–	–	–
*Coenonympha arcania*	*Cacr*	NT	+	+	+	+	+	+	+	+
*Coenonympha glycerion*	*Cgly*	–	+	+	–	–	+	+	+	+
*Coenonympha pamphilus*	*Cpam*	–	+	+	+	+	+	+	+	+
*Erebia medusa*	*Emed*	NT	+	–	–	–	–	+	+	+
*Erebia aethiops*		EN	+	–	–	–	–	–	–	–
*Melanargia galathea*	*Mgal*	–	+	+	+	+	+	+	+	+
*Lasiommata megera*	*Lmeg*	–	+	+	+	+	+	+	+	+
*Lasiommata maera*		NT	+	–	–	–	–	–	–	–
*Pararge aegeria*^$^	*Paeg*	–	+	+	–	–	–	–	+	+
*Maniola jurtina*	*Mjur*	–	+	+	+	+	+	+	+	+
*Hyponephele lycaon*		CR	+	–	–	–	–	–	–	–
*Aphantopus hyperanthus*	*Ahyp*	–	+	+	+	+	+	+	+	+
*Hipparchia semele*		CR	+	–	–	–	–	–	–	–
Lycaenidae										
*Thecla betulae*		–	+	–	–	–	–	–	–	–
*Neozephyrus quercus*^$^		–	+	–	–	–	–	–	–	–
*Callophrys rubi*	*Crub*	NT	+	+	+	+	+	+	–	+
*Satyrium acaciae*	*Saca*	–	–	–	–	–	–	–	+	+
*Satyrium pruni*	*Spru*	NT	+	–	–	–	–	+	–	+
*Satyrium w-album*^$^		NT	–	–	+	–	+	–	–	–
*Satyrium spini*	*Sspi*	VU	–	–	–	–	–	–	+	+
*Lycaena alciphron*		VU	–	–	–	+	+	–	–	–
*Lycaena dispar*	*Ldis*	–	–	–	–	–	–	+	+	+
*Lycaena phlaeas*	*Lphl*	–	+	+	+	+	+	+	+	+
*Lycaena tityrus*	*Ltit*	–	+	+	+	+	+	+	–	+
*Lycaena virgaureae*	*Lvir*	NT	+	+	+	+	+	+	+	+
*Celastrina argiolus*	*Carg*	–	+	+	+	+	+	+	+	+
*Cupido minimus*	*Cmin*	VU	+	+	–	–	+	+	+	+
*Aricia agestis*	*Aage*	–	+	+	+	+	+	+	+	+
*Aricia eumedon*		NT	+	–	–	–	–	–	–	–
*Plebejus argyrognomon*	*Pargy*	–	+	–	–	–	–	+	+	+
*Plebejus argus*	*Parg*	NT	+	+	+	+	+	+	+	+
*Cyaniris semiargus*		VU	–	–	–	+	+	–	–	–
Phengaris alcon*	*Palc*	EN	+	+	+	+	+	+	+	+
*Polyommatus bellargus*	*Pbell*	VU	–	–	–	–	–	–	+	+
*Polyommatus amandus*	*Pama*	NT	+	+	+	+	+	+	+	+
*Polyommatus icarus*	*Pica*	–	+	+	+	+	+	+	+	+
*Polyommatus coridon*	*Pcor*	VU	+	+	+	+	+	+	+	+
*Polyommatus daphnis*	*Pdap*	VU	+	+	+	+	+	+	+	+
*Polyommatus thersites*		VU	+	–	–	–	–	–	–	–
Total		32	71	50	47	49	51	55	55	60
Arboreal + migrants excluded		31	61	44	45	42	46	50	50	54

(NT—near threatened, VU—vulnerable, EN—endangered, CR—critically endangered) following [46]. The early 1990s data are from [62], 2009 data from [23], and this study to the 2016–2019 monitoring. Abbreviations are used in the ordination diagram at Fig. 4

^$^Arboreal and ^$$^migrant species excluded from some analyses

^*^The “*rebeli*” ecological form, developing on *Gentiana cruciata*

**Fig. 2:**
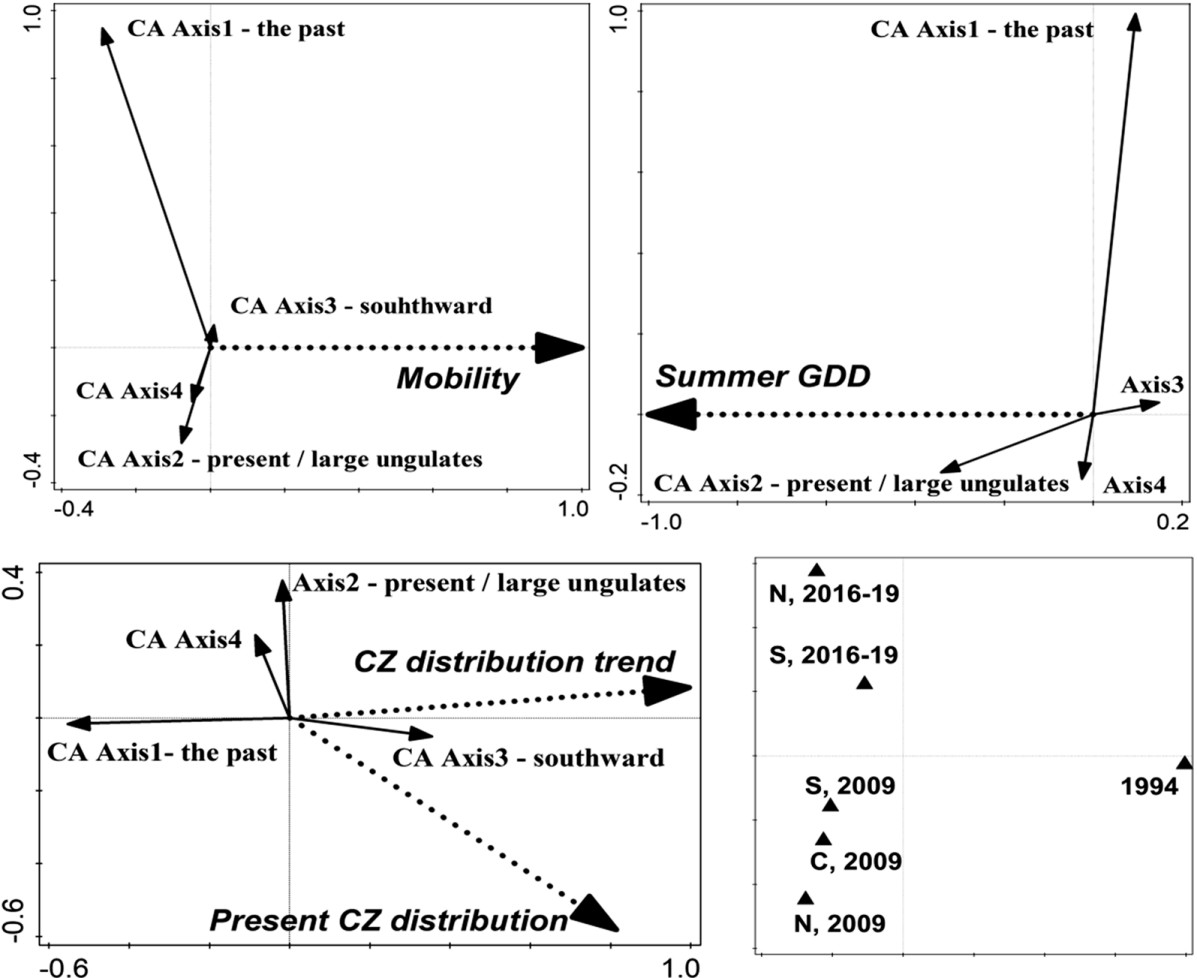
Interpreting three subsequent butterfly surveys of the Milovice military range by species traits. Results of CA analysis of presence/absence data obtained from the Milovice military training range (six-level analysis, arboreal and migrant species excluded: Table 2) interpreted by life history traits (top left), climatic niche traits (top right) and conservation attributes (bottom left) of constituent species. Positions of the three surveys, based on CA ordination of recorded butterflies, in bottom right

The indirect CA analyses (Fig. 2) revealed differences among the three surveys in butterfly species composition (total variation = 0.22, axis 1 separating the earliest and the two subsequent surveys: 59.5%, axis 2 distinguishing the interim and the current survey: 40.5%). The pattern held if the localities N, C, S were treated separately (variation = 0.40; % subsequent axes: 45.2, 29.0). Removing 11 arboreal and migrant species (cf. Table 1) decreased the explained variation (three samples variant: 0.25, six samples variant: 0.35) without changing the overall pattern (% successive axes: 59.9, 40.1; 47.9, 31.0).

Interpreting the CA ordinations by species` attributes gave results consistent across the four variants (Table 2). Species present shortly after the cessation of military use and lost subsequently tended to be less mobile. Their ranges were characterised by a broader oceanity niche, narrower continentality niche and lower mean annual temperatures (Fig. 2). Species inclining towards the current survey require higher mean annual temperatures and higher numbers of growing degree days. The species lost since the earliest survey are declining in the Czech Republic, while those gained recently display rather restricted distributions in the country (Table 2).

**Table 2:** Results of explaining species scores obtained from the correspondence analyses (CA) of three successive butterfly assemblages surveys (early 1990s, 2009, 2016–19) in the (former) Milovice military training range, by life history traits, climatic niche traits and conservation attributes of constituent species. See Table 5 for explanation of the traits

	Life history traits				Climatic niche traits				Conservation attributes			
Analysis	Ordination axes: traits correlations	% var	Axis1 F, P	All axes F, P	Ordination axes: traits correlations	% var	Axis1 F, P	All axes F, P	Ordination axes: traits correlations	% var	Axis1 F, P	All axes F, P
3-level		–	–	–	** + Ax1**: Oceanity NB; **−Ax1**: Summer GDD NB ** + Ax2**: Early summer GDD, Summer GDD **−Ax2**: Summer GDD NB	17.0	14.5***	5.0***	**−Ax1**: Distribution trend CZ; Current range CZ **−Ax2**: Current range CZ	17.3	19.9***	9.2***
3 level, arboreal/migrant spp. excluded	**−Ax1**: Mobility	2.2	2.5 +	–	** + Ax1**: Precipitation NB **−Ax1:** Early summer GDD ** + Ax2**: Continentality NB	13.5	10.9**	4.5***	**−Ax1**: CZ distribution trend, CZ current range; ** + Ax2**: CZ current range	20.8	13.4***	9.8***
6-level	**−Ax1:** Mobility	2.0	2.6*	–	**−Ax1**: Summer GDD ** + Ax1:** Summer GDD	2.5	3.0*	–	**−Ax1**: CZ distribution trend, CZ current range; **−Ax2**: CZ current range	18.6	15.9***	12.5***
6 level, arboreal/migrant spp. excluded	**−Ax1**: Mobility **−Ax2**: Mobility	3.5	3.4*	–	**−Ax1**: Summer GDD ** + Ax2**: Summer GDD	2.4	2.6*	–	**−Ax1**: CZ distribution trend, CZ current range; **−Ax2**: CZ current range	20.2	14.7***	9.5***

3-level analyses pooled individual sites surveyed, while 6-level analyses treated the grasslands sites S, C, and N separately, if allowed by the data. −/ + signs preceding the ordination axes Ax1–Ax4 values indicate the direction of the correlation with respective CA axes. **%var, F** and **P** values refer to Monte Carlo tests for the significance of the relationships between trait values and CA ordination scores

### Current monitoring of refaunation effects

The 61 species currently recorded (Table 1, Additional file 2) were observed in 25,322 individuals. The mean (± SD) /median/ range per plot and year, summed across the five yearly visits, were 24.6(± 4.88)/ 24/ 15–38 species, and 395.7(± 214.55)/ 343/ 99–1,057 individuals. Species and individuals’ numbers were positively correlated (Pearson’s r = 0.511, t_(N=64)_ = 4.61, P < 0.0001).

**Fig. 3:**
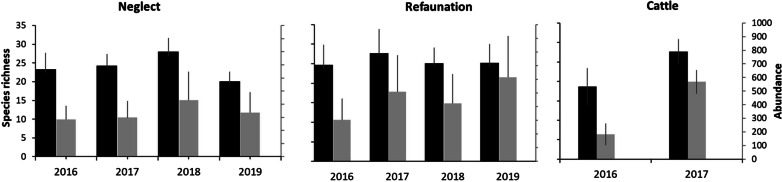
Butterfly species richness and abundance of neglected and refaunated plots. Numbers of butterfly species (black bars) and individuals (grey bars) recorded in the former Milovice military training range, with respect to management of the plots. Means ± SD recorded per the plot and year are shown

The numbers of species per plot differed significantly among years and refaunated plots hosted more species (mean: 25.7 ± 4.96SD) than neglected ones (23.5 ± 4.50SD), except for 2018 with an opposite pattern, resulting into marginally significant management x year interaction (mixed linear model, year F = 2.83 (df: 3, 45.04), P < 0.05; management F = 4.41 (df: 1, 44.60), P < 0.05; interaction F = 2.60 (df: 3, 45.09), P = 0.06). The numbers of individuals did not differ among years (F = 2.00 (df: 3, 45.54), P = 0.13) and were consistently higher at refaunated (457.4 ± 251.21SD) than at neglected (345.8 ± 166.75SD) plots (management F = 15.07 (df: 1, 45.54), P < 0.001; interaction F = 1.76 (df: 3, 45.03), P = 0.17). At the cattle-grazed plots, the numbers varied highly from year to year (Fig. 3).

In the CCA analyses (Table 3), the covariates nectar, hour, and weather did not affect the composition of assemblages, implying that nectar was available rather evenly across the plots and visits, and visits were mostly under suitable weather. The strong effect of factorially coded year explained the highest variation of all (co)variables. It was followed by plots position, specifically latitude, collinear with the effect of site. Tanks as a separate predictor had no effect. Refaunation alone had no significant effect. For ungulates, the first axis, which distinguished aurochs and horse from neglect, cattle and wisent, was marginally significant. The marginally significant second and (still canonical) third axes distinguished neglect and cattle, respectively, from other situations. Adding tanks into either ungulates or refaunation models increased the models’ statistical significance, suggesting that some butterflies responded to the thus created intensive disturbance.

**Table 3:** Results of CCA analyses, comparing the 2016–19 current monitoring results from plots refaunated by large ungulates versus neglected plots

Model	E1	E2	E3	E4	% variation	Axis1 F, P	All axes F, P
~ Nectar	0.039				1.2	4.9, ns	
~ Weather	0.034	0.027			1.7	4.2, ns	3.8, ns
~ Factorial hour	0.021	0.016	0.012	0.008	0.6	2.5, ns	1.3 +
~ Polynomial hour	0.017	0.006			0.3	2.1, ns	1.4*
~ Factorial year	0.114	0.049	0.019		6.1	14.5*	7.9**
~ Linear year	0.045				1.4	5.6, ns	
~ Position (forward selected: latitude)	0.026				0.7	3.2**	
~ Site	0.026				0.7	3.2**	
FW covariate model^a^ (~ factorial year + latitude)	0.114	0.054	0.020	0.019	8.0	14.5***	6.8***
BW covariate model^b^ (~ factorial year + latitude + nectar)	0.120	0.056	0.035	0.019	8.1	15.3***	6.6***
~ Tanks	0.015				0.3	1.9, ns	
~ Tanks | factorial year + latitude	0.007				0.1	1.0, ns	
~ Tanks | factorial year + latitude + nectar	0.007				0.0	1.0, ns	
~ Refaunation^c^	0.019	0.010			0.5	2.3, ns	1.8, ns
** ~ Refaunation | factorial year + latitude**	**0.019**	**0.006**			**0.4**	2.5**	1.7**
** ~ Refaunation | factorial year + latitude + nectar**	**0.016**	**0.006**			**0.3**	2.5*	1.5*
~ Refaunation + tanks	0.029	0.014	0.010		1.1	3.6, ns	2.2*
** ~ Refaunation + tanks | factorial year + latitude**	**0.022**	**0.009**	**0.004**		**0.5**	2.9**	1.6**
** ~ Refaunation + tanks | factorial year + latitude + nectar**	**0.023**	**0.008**	**0.004**		**0.6**	3.1**	1.6**
~ Ungulates^d^	0.023	0.015	0.010	0.005	0.8	2.8 +	1.6 +
** ~ Ungulates | factorial year + latitude**	**0.029**	**0.010**	**0.009**	**0.004**	**0.4**	3.0*	1.3*
** ~ Ungulates | factorial year + latitude + nectar**	**0.019**	**0.009**	**0.006**	**0.003**	**0.3**	2.6*	**1.2 + **
~ Ungulates + tanks	0.035	0.015	0.015	0.010	1.4	4.2*	1.9*
** ~ Ungulates + tanks | latitude + factorial year**	**0.023**	**0.010**	**0.009**	**0.004**	**0.4**	3.0*	1.3*
** ~ Ungulates + tanks | latitude + factorial year + nectar**	**0.025**	**0.009**	**0.008**	**0.005**	**0.5**	3.4*	**1.3 + **

Bold E1–E4 are eigenvalues of respective canonical axes, bold F and P values refer to results of Monte Carlo tests for the first canonical axis and all canonical axes. The models written in **bold** were used for interpreting the ordination axes by species traits (see Table 4)

^a^Obtained by forward selection (FW) from all significant terms above

^b^Obtained by backward selection (BW) from all terms above

^c^3-level factor (refaunation, cattle, and neglect)

^d^5-level factor (horse, aurochs, wisent, cattle, and neglect)

^+^P < 0.1, ^*^P < 0.05, ^**^P < 0.01, ^***^P < 0.001

**Fig. 4:**
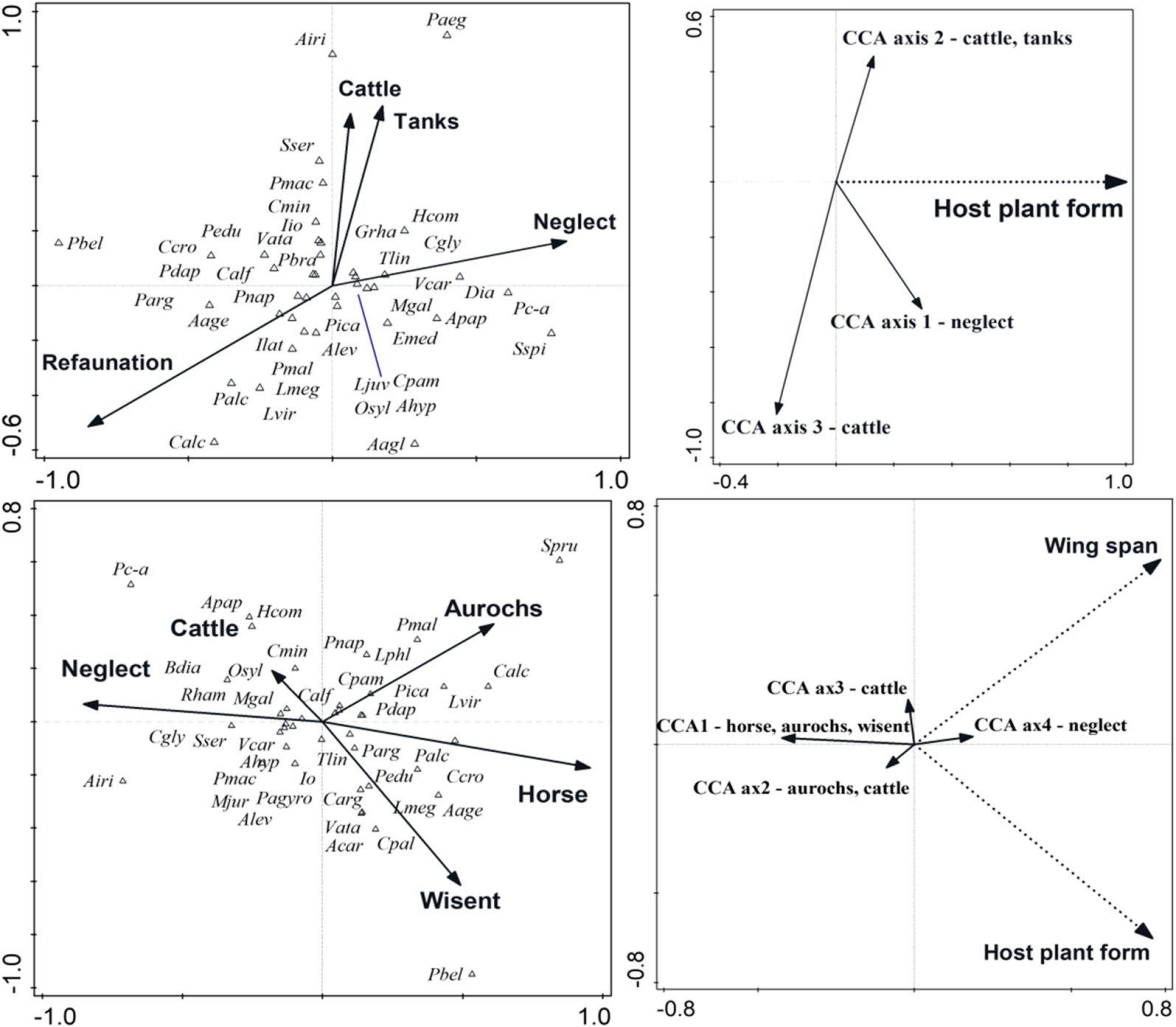
Ordination analysis of large ungulates refaunation effects on butterfly assemblages, and interpreting the results by species traits. Top left: CCA biplot relating the current (2016–19) butterfly species composition at monitored plots in the Milovice former military training area to refaunation (model (~ refaunation + tanks | factorial year + latitude; i.e., FW selected covariables). Top right: RDA biplot interpreting the ordination axes by species traits (details: Table 3). Bottom left: CCA biplot for model (~ ungulates | factorial year + latitude + nectar, i.e., BW selected covariables). Bottom right: RDA biplot interpreting the CCA axes by traits. See Table 3 for CCA models parameters and Table 4 for RDA models parameters

The models much improved after inclusion of FW (factorial year + latitude) and BW (factorial year + latitude + nectar) selected covariates (Table 3, Fig. 4). This implied that the effects of the focal predictors were originally masked by the variation among years and collinearity between grazing regimes and the plots` position.

For refaunation, the ordinations now separated plots grazed by large ungulates from neglected plots (first axis), and cattle plus tanks from all the other regimes (second axis). The butterflies closely associated with large ungulates were narrowly specialised forbs feeders, such as the obligatorily myrmecophilous *Phengaris alcon*, multiple other Lycaenidae (*Plebejus argus*, *Polyommatus coridon*), but also some Pieridae (*Colias alfacariensis*) and Hesperiidae (*Erynnis tages, Pyrgus malvae*). Species associated with neglect were those preferring coarse grasslands (the fritillary *Boloria dia*; the Satyrinae *Melanargia galathea*, *Maniola jurtina*; the hesperids *Ochlodes venatus*, *Hesperia comma*) and shrubs (*Iphiclides podalirius*, *Coenonympha arcania*). Cattle pasture was associated with common generalists (*Pieris brassicae*, *Vanessa cardui*, *Thymelicus lineola*), but also with the nationwide vulnerable hesperid *Spialia sertorius*, which was also closely associated with *tanks*. (See Additional file 3 for positions of all butterfly species.)

Almost identical patterns arose in the analysis with *ungulates*. The first axis distinguished neglect from the three megafaunal species, the second axis distinguished cattle plus tanks, and the (still canonical) third and fourth axes separated aurochs from wisent, and cattle from other management types, respectively (Table 3, Fig. 4, Additional file 3).

Interpreting the resulting CCA models by species traits (Table 4, Fig. 4, Additional file 4) consistently related both refaunation and wild ungulates presence either to host plant form, or to wing span, or to both. Butterflies inclining towards neglect tended to develop on woody plants or coarse grasses and/or tended to be larger than those inclining towards refaunation / ungulates. No climatic niche or conservation-related attribute performed significantly in these analyses.

**Table 4:** Interpreting results of selected CCA models, comparing the 2016–19 current monitoring results from plots refaunated by large ungulates versus neglected plots (cf. Table 3), by species traits. Species ordination scores from the CCA were related to life history, climatic niche and conservation related traits via redundancy analysis (RDA), best-fitting combinations were forward-selected. See Additional file 4 for single effects of all traits to all models assessed

Model	% variation	E1	E2	Axis1 F, P	All axes F, P	
~ Refaunation | factorial year + latitude	5.5	0.070		4.5*		Host plant form—small forbs with refaunation, bulky plants towards neglect
~ Refaunation | factorial year + latitude + nectar	4.4	0.060		4.4*		Host plant form—as above
~ Refaunation + tanks | factorial year + latitude	2.4	0.040		2.5 +		Host plant form—as above
~ Refaunation + tanks | factorial year + latitude + nectar	4.4	0.060		3.8*		Host plant form—as above
~ Ungulates | factorial year + latitude	2.5	0.042		2.6 +		Host plant form—as above
~ Ungulates | factorial year + latitude + nectar	2.1	0.037		2.3 +		Wing span—increasing towards neglect
~ Ungulates + tanks | latitude + factorial year	1.7	0.034		2.1 +		Host plant form, small forbs with wild ungulates, bulky plants towards neglect
~ Ungulates + tanks | latitude + factorial year + nectar	3.1	0.056	0.007	3.4 +	2.0 +	Host plant form—as above Wing span—increasing towards neglect

^+^: P < 0.1, ^*^: P < 0.05

## Discussion

The former Milovice military training area harbours rich butterfly assemblages; the 55–60 species currently recorded per site is above average for nature reserves in the country [4, 23, 83]. This richness was arguably preserved there owing to exclusion of intensive agriculture and forestry, combined with the past finely-grained disturbance-succession dynamics typical for military areas [15, 23, 76]. Following the cessation of military use, several species were lost, while others were subsequently gained. Presence of aurochs, horses, and wisents increases per-plot butterfly species richness and abundance, presumably by manipulating vegetation conditions, thus supporting multiple species of conservation concern, e.g. the critically endangered obligatorily myrmecophilous *Phengaris alcon* [cf. 91].

### Changes since termination of military use

The termination of military activities was followed by the successional overgrowth of the disturbed sparsely vegetated surfaces, and gradual dominance of coarse grasses and tall forbs [52]. We therefore expected (hypothesis H1) decrease of specialists associated with small competitively inferior forbs, which our analyses of life history traits did not support. The only life history trait responding to the past-present ordination was mobility. Poorly mobile species were associated with the past military use. Among European butterflies, high mobility is a generalist trait associated with broad trophic ranges, long flight period and other features facilitating survival in human-dominated landscapes [5, 26, 45], whereas poor mobility increases extinction risks [12, 33]. Because mobility relates inversely to local population density [4], some poorly mobile species may need large habitat areas to sustain viable populations. The changes after cessation of military use probably led to shrinking habitats supply for poorly mobile specialists.

Associations of lost and gained species with climatic niche traits (H2) were more straightforward. In agreement with the warmer and drier climate in Central Europe during the last few decades [87], the locally lost species shared broad oceanity or precipitation niches, whereas the newly gained species require higher temperatures. Also, in agreement with H3 stating that species newly colonising the area should be those currently increasing in distribution, the lost species display decreasing distribution trends in the Czech Republic and elsewhere in Central and Western Europe, whereas the opposite applies for the newly gained species [cf. 100].

A combination of restricted mobility and broad oceanity or precipitation niches applies to several locally lost and nationally threatened species [cf. 9,47]: the hesperids *Pyrgus armoricanus* (currently re-expanding elsewhere in Central Europe [11, 57]) and *Thymelicus acteon*, and the satyrines *Hipparchia semele*, *Hyponephele lycaon*, and *Erebia aethiops*. The latter is a sparse woodland species [82] only loosely associated with grasslands, but its current occurrence in the area was safely excluded by concurrent targeted searches. The remaining four, all declining in Central Europe [100], require sparsely vegetated substrates and often colonise such landforms as disused quarries and post-industrial barrens [8, 13, 95,96]. Broad oceanity tolerance certainly applies to *Hipparchia semele*, distributed from Eastern Europe to Atlantic coastal dunes [78], but also to *Hyponephele lycaon* and *Pyrgus armoricanus*, whose ranges follow maritime climates far north to southern Fennoscandia [35, 66]. The species newly gained during the last two decades include *Iphiclides podalirius*, *Satyrium acaciae*, *S. spini*, *Lycaena dispar*, and *Polyommatus bellargus*, all currently (re)expanding in Central Europe. The first three are associated with shrubs [9], and the fourth with tall ruderal forbs [86], hence they might have profited from concurrent effects of successional abandonment. Only the fifth, gained as late as 2018, develops on *Securigera varia* (L) host plants growing at sparsely vegetated surfaces [8], likely profits from the ungulates’ grazing. The gains and losses thus reflect the interaction of local management changes, and forces affecting species pool at larger scales [75, 93].

### Refaunation by large ungulates

Plots affected by large ungulates displayed increased butterfly species richness and abundance, supporting our hypothesis H4 that wild ungulates grazing should increase butterfly resource base [40, 55]. At the same time, refaunation affected the local assemblages’ composition. It favoured smaller species developing on small forbs over larger species developing on large forbs, grasses or shrubs, supporting our hypothesis H5.

As in other studies [25, 49, 108], the immediate effects of year-round ungulates’ presence included reduction of tall coarse grasses, slowing down scrub growth due to browsing and bark peeling, reduction of grass blooming by consuming grass inflorescences, and exposing barren ground around tracks and wallows. As in experiments with feral horses [40], some richly blooming forbs, including species that rarely bloomed in the years preceding the refaunation, increased in abundance [31]. The differences in butterfly assemblages` composition between refaunated and neglected plots became more apparent after statistical control for the effect of year and the monitored plots position. Still, species benefitting from refaunation included the iconic *Phengaris alcon f*. *rebeli*, whose host plant, the poorly competitive [cf. 43,71] and chemically protected [73] perennial *Gentiana cruciata*, boomed shortly after the establishment of grazing. This obligatorily myrmecophilous butterfly is likely host plant limited, because its females prefer oviposition on prominent plants overtopping surrounding vegetation [44, 64, 103]. Another limitation may be presence of symbiotic *Myrmica* sp. ants, and the ungulates-ants dynamics deserves more detailed research [91].

Interpretations of the assemblages’ responses by species attributes risk being confounded by phylogeny, because life history and even climatic niche traits are often phylogenetically conserved [20]. The traits used in our analyses, however, were shown to retain basic topology of their mutual relations when controlled for phylogeny [5], and the species developing on small herbs and favoured by refaunation belong to several families, suggesting robustness of this result.

The simplest explanation of the higher butterfly richness and abundance at refaunated plots, congruent with the positive richness—abundance correlation, is the generally smaller size of the forbs-feeding specialists, a trait known to be associated with higher local population densities and lower mobility [4, 5, 26]. In European butterflies, large body size is also associated with lower number of generations and feeding on mechanically, rather than chemically, protected host plants, i.e., woody species and grasses, favoured by more advanced succession [22] but supressed by ungulates presence. In contrast, many plant groups avoided by horses (e.g., Rosaceae, Fabaceae, Polygonaceae, Orobanchaceae: [21]), once the dominant grazers of West-Palaearctic grasslands, are frequent in the larval diet of European butterflies. Possible coevolutionary relationships between mammalian megafauna and herbivorous insects, and their conservation implications, deserve further investigation.

The patterns revealed by ordinations relating species composition to refaunation were admittedly less convincing than in studies comparing starkly contrasting habitats, such as close woodlands vs. clearings [e.g., 10,80]. It appears that the refaunated and neglected plots were interconnected by individual movements. The distances among study plots were within the routine movement abilities of most butterflies [36, 85], although this may not apply for the least mobile species [58]. Also, the small-scale vegetation mosaic at the study sites ([52]; Fig. 1) could blur potential effects to species community structures. It is likely that individual butterflies located some of their vital resources at both grazed and ungrazed sections of the area, in line with the resource-based understanding of animal habitats [17, 97].

The setting of our study did not allow distinguishing between the effects of horses and big bovids, as both pastures contained combinations of these ungulates. The literature on refaunation in temperate [e.g., 102,108] and northern boreal [61] regions agrees that these two ungulate groups supplement each other in effects on vegetation, as well as seasonal and diurnal habitat use. Additionally, both horses and bovids acted as dominant grazers in late Quaternary European ecosystems, and both were present as domesticated forms in traditional rural landscapes.

The mechanical disturbance by armoured vehicles (factor tanks) exhibited no separate effect, seemingly countering the claims [48] that it provides disturbed conditions beneficial for some insects. Presence of tanks, however, increased the explanatory power of models containing ungulates or refaunation effects (Table 3), suggesting a complementarity with large grazers for some butterfly species. This might be the case of *Spialia sertorius*, a skipper associated with tanks in ordination diagrams and developing on *Sanguisorba minor*, a competitively inferior forb preferring sparsely vegetated surfaces [42]. Arguably, on military lands, and in the current Milovice reserves, the heavy vehicles supplement yet another lost component of the megaherbivore fauna of interglacial Europe, proboscideans [99].

The effect of domestic cattle, grazed at three plots for two years of the project, was orthogonal to the ordination gradient distinguishing refaunation and neglect. The cattle were grazed with high stocking and supplementary feeding during the vegetation season and were not present in winter. Such grazing style suppresses forbs and fails to suppress coarse grasses. Grazing by domestic breeds in more biodiversity-friendly ways is possible [32, 46, 49], but this was not the case in our system.

While being demonstrably positive for butterflies associated with poorly competitive forbs, the refaunation did not detectably imperil species associated with coarse grasses or shrubs. In this respect, the Milovice situation differs from some projects with documented negative outcomes for insect assemblages [98]. It seems beneficial that contrary to some refaunation sites amidst urbanised landscapes [60], our study system is situated in a diverse rural setting, including ungrazed/neglected plots, which provide conditions contrasting with the grazed sites. This habitat diversity likely allows for resource compensation/supplementation by the butterflies [69], enabling coexistence of species requiring different disturbance levels [18]. The current grazing pressure ≈0.5 grazers*ha^−1^ does not deplete the sites of larval host plants or nectar. There is a potential long-term risk, as the whole operation is funded from the EU Agri-environmental scheme “grazing”, which requires maintaining stable grazing intensity. Flexibility may be necessary, as grazing levels appropriate for restoring overgrown sites may become too high once species-rich dry grasslands develop, as well as if accelerating climate change will decrease rainfall levels during the vegetation period.

## Conclusions

Whereas abandonment and successional changes of a former military area restructured the rich local butterfly fauna, refaunation of parts of the area by megafaunal grazers contributes to maintaining high butterfly species richness and abundance. Analysing traits of the constituent butterfly species revealed that the post-abandonment changes, spanning across two decades, affected butterfly assemblages via different mechanisms than does the current megaherbivores activity. The post-abandonment changes led to losses of some poorly mobile species and gains of some regionally expanding species, presumably rather good dispersers. The changes also had a climatic component, indicated by differences in climatic niche traits between past and present assemblages. At present, the megaherbivores affect butterfly assemblages by transforming vegetation, and hence supporting smaller species developing on small forbs on the expense of larger species developing on bulky forbs, coarse grasses, and woody plants. Local heterogeneity of conditions, and existence of ungrazed sections in the vicinity of the grazed ones, ensure that species from the other group are not locally imperilled. Given that many of the species lost since abandonment of the area by the military were poor dispersers, reintroductions of some of the lost species, whose habitats the ungulates have restored, is a logical next step.

Unresolved questions include differences among ungulate species in affecting butterfly larval and adult resources, possible legacies of coevolution between temperate butterflies and ungulates, and future development of the butterfly assemblages. The latter question is tractable by sustained monitoring, whereas the former two can be approached by expansion of studies similar to ours to sites varying in composition of both butterfly assemblages and ungulate species. This ambitious programme is increasingly feasible, as the refaunation movement expands and the number of potential study systems rapidly increases. In the Czech Republic alone, progeny of the Milovice ungulate herds currently roam at an additional seven sites, offering rich opportunities for future research.

## Methods

### Study area, refaunation, and earlier butterfly surveys

The Milovice military training range (50.26 N, 14.89E, altitude 200–250 m a.s.l., mean annual temperature 8–9 °C, annual precipitation 500–600 mm) (Fig. 1) was established in 1904, originally on 34.6 km^2^. It was subsequently used by all armies that operated on Czech territory, gradually expanding its area to 40 km^2^. The last users were the Soviets, who operated an air force base and headquarters here for the former Czechoslovakia until 1991. The natural setting is the gently rolling Středočeská Tabule Plain formed by Mesozoic carbonate-rich sandstones, siltstones, and claystones, and covered by brown soils, rendzinas, and carbonate rich sands. Woodlands dominated by *Quercus petraea*, *Pinus sylvestris*, and *Betula pendula* are interspersed by finely grained mosaics of shrublands, grasslands, and early successional vegetation that developed on former farmlands (mainly wheat, vegetables, and dairy family farms) and were utilised for training troops for over 80 years [23, 52].

Following the cessation of military use, parts of the open training fields were developed (golf course, amusement park, industrial zone), while three large areas were proclaimed a Site of European Community Importance (SCI) Milovice-Mladá. The Central site (local toponym: Pozorovatelna, hereinafter “C”, 50.254 N, 14.881E) has been partly managed by conservation grazing by fenced sheep, while the Northern (Traviny, “N”, 50.278 N, 14.883E) and Southern (Pod Benáteckým vrchem, “S”, 50.241 N, 14.886E) sites remained unmanaged, except for occasional disturbance of S by armoured vehicles practiced by military history enthusiasts and for domestic cattle grazing in a corner of N in 2014–2016. Much of all three sites had suffered succession-driven homogenisation of the once diverse vegetation mosaic by competitively dominant grasses (mainly *Calamagrostis epigejos* and *Arrhenatherum elatius*), ruderal forbs and shrubs (mainly *Crataegus*, *Prunus*, and *Rosa*).

The site S (2015–2017, 40 ha; 106 ha since 2018) has been grazed since spring 2015 by ≈35 Exmoor ponies (hereinafter “horse”) and ≈20 Tauros cattle (hereinafter “aurochs”). Since spring 2016, ≈35 horses and ≈20 wisents have grazed the site N (125 ha) (Fig. 1). Both S and N are thus year-round cross-grazed by horses and big bovids (aurochs or wisent) living in naturally structured social units, i.e. mixed sex/age harems/herds. To provide variable management regimes, both temporally and permanently ungrazed plots of various sizes (units to tens of hectares) are present both within and outside the grazing reserves at any given time. The animals receive no supplementary feeding and no medication, except for strictly determined individual cases, and predators enter the sites freely [54]. The wolf, as a re-expanding apex predator, is not present yet, but its colonisation is expected. To control grazing intensity, facilitate gene-flow, and avoid social stress, two to three year-old surplus animals are transferred to similar projects in the Czech Republic and abroad.

The first targeted butterfly survey of the area was conducted in the early 1990s, immediately after the cessation of military use. It produced a commented list of species, treating the entire military range as a single locality [62]. Fifteen years later, in 2009, the training fields S, C and N were surveyed separately in a semiquantitative manner, recording maxima per visit at a logarithmic scale [23]. The current monitoring of the refaunation impact, launched in spring 2016, thus represents the third survey.

### Current butterfly monitoring

We set 16 rectangular plots (50 × 200 m) at both refaunated (n = 7) and neglected (n = 9; three of them grazed by cattle for two years) sections of N and S sites (n = 8 each) (Fig. 1). In 2016–2019, one of us (DR) visited the plots five times each year (May, early June, late June, July, August) to cover seasonal aspects of butterfly assemblages. The recording followed the timed survey protocol [56], appropriate for heterogenous environments with temporally changing locations of butterfly resources, such as flower patches. Each visit to a plot lasted 30 min, abundances of all butterfly species observed were recorded using a net when necessary and taking vouchers of species not recognisable in the field. We also recorded the closest hour, cloudiness (3-point ordinal scale, from clear sky—1 to overcast—3), wind (Beaufort scale 1—4, i.e., calm to gentle breeze), and nectar supply (0—no flowers within the plot, 1—flowers scarce but present, 2—flowers moderately abundant, 3—flowers abundant). We restricted the visits to the highest butterfly activity period (10 AM—4 PM) and to weather suitable for butterflies, randomising their sequence with respect to time of day. A single round of visits took 2–3 consecutive days.

### Past-present comparison

For the past-present comparison, we visualised the patterns defined by species presences/absences recorded in early 1990s [62], 2009 [23], and during the current monitoring, the latter collated across the four years, using correspondence analysis (CA), an unconstrained ordination appropriate for 1/0 data, in CANOCO, v. 5.0 [89]. We computed four variants of CAs: (1) based on three “samples” defined by the three consecutive surveys; (2) differentiating records from the locations N, C, and S (possible using [23] and the current data), thus obtaining six “samples”; and (3 + 4) as in the previous two cases, but after exclusion of migrant and arboreal species.

We interpreted the CA results by three sets of the constituent species` attributes (Table 5, Additional file 1): (a) Life history traits, as compiled for Central Europe [5]. This selection of traits associated with feeding modes, dispersal and population structure reveals a generalist-specialist continuum in the butterfly fauna [cf. 19,65], while also distinguishing multivoltine species associated with small ruderal forbs from univoltine species associated with trees, shrubs and grasses [2]. (b) Climatic niche traits, compiled in [79] on the basis of species ranges in Europe and known to contribute to population trends [33]; and (c) Conservation attributes describing the distribution and Red-list status in the Czech Republic. We used the CANOCO option “explanation of species scores for functional traits”. This analysis, a multivariate version of the fourth-corner approach [30, 59], relates the species ordination scores from the CA ordination to trait values of the species, testing for strengths of the relationship using redundancy analysis (RDA), a multivariate version of linear regression [89]. We analysed the three sets of traits separately, using the forward selection process to attain best-fitting traits combinations.

**Table 5:** Life history traits, climatic niche traits and conservation-related attributes used for analyses of butterfly assemblages inhabiting the former Milovice military range, refaunated by large ungulates

	Description	Character
*Life history traits *[5]		
Wing span		Numeric (mm)
Host plant form	Ephemerals—1, larger forbs—2, grasses and sedges—3, trees and shrubs—4	Ranked
Voltinism	Average number of generations, C. Europe	Numeric
Fertility	Average number of eggs per female at eclosion	Categories 1–9
Mobility	Ranked tendency to disperse	Categories 1–9
Density	Ranked average density per area of habitat	Ranked 1–9
Diet breadth	Number of plant families fed on by larvae in the Czech Republic	Numeric
Flight period length	Number of adult occurrence months (hibernation months excluded)	Numeric
Overwintering stage	Ranked, larva—1, adult—5	
*Climatic niche traits *[79]		
Annual temperature	Mean annual temperature	°C
Annual temperature niche breadth [= NB]	SD of the above	
Continentality	Annual range in monthly temperatures	°C
Continentality NB	SD of the above	
Precipitation	Annual precipitation sum	mm
Precipitation NB	SD of the above	
Oceanity	Annual range in monthly precipitation sum	mm
Oceanity NB	SD of the above	
Winter GDD	Accumulated growing degree days [GDD] (> 5 °C), January–February	°C
Winter GDD NB	SD of the above	
Spring GDD	Accumulated GDD, January–April	°C
Spring GDD NB	SD of the above	
Early summer GDD	Accumulated GDD, January–June	°C
Early summer GDD NB	SD of the above	
Summer GDD	Accumulated GDD, January–August	°C
Summer GDD NB	SD of the above	
Water availability	Soil water content of the upper horizon (0.5 m)	No unit (0–1)
Water availability NB	SD of the above	
*Conservation attributes*		
Red list status [47]	Czech Republic (1—no status, 2—near threatened, 3—vulnerable, 4—endangered, 5—critically endangered)	ranked 1–5
Present CZ distribution [5]	Occupied Czech Republic 10 × 10 km grid squares 2002–2014	Numeric
Past CZ distribution [5]	Occupied Czech Republic grid squares 1951–2001	Numeric
CZ distribution trend [5]	[1- (Present distribution/Past distribution)]	Numeric
Global range size [5]	Categorized, (1—smaller than Europe, 5—larger than the Palaearctic)	Numeric
European range size [79]	Number of occupied (ca 70 × 70 km) grid squares in Europe	Numeric

### Current monitoring

To compare numbers of butterfly species and individuals recorded, we used linear mixed-effects model in the R package lmer4 with Nelder-Mead optimisation parameter [7]. The factors *year* (4 levels), *management* (2 levels, refaunation *vs*. neglect) and their interaction were fixed, whereas the identity of site (i.e., the pastures N and S) was entered as a random factor. This approach partly ameliorated the problem with non-independence of plots within the two pastures. The three plots grazed by cattle in 2016–17 were excluded from this analysis for these years.

To study the refaunation effects on the per-plot composition of butterfly assemblages, we used canonical correspondence analysis (CCA), a constrained ordination method relating the species composition of samples to external predictors and testing the relationships of species composition to predictors using the Monte Carlo test (999 permutations), again in CANOCO. We log-transformed species abundances per plot visits, and downweighted rare species (a default option). We reflected the temporal structure in the data using a hierarchical permutation design, permuting the individual plots randomly, and the 20 subsequent visits per plot as mutually dependent cyclic shifts.

We first ran CCAs for the pivotal effect of refaunation, for which we used two different codings, targeting two related questions: Refaunation (3-level factor: refaunation, neglect, and cattle) aimed on the effect of wild ungulates presence, whereas Ungulates (5-levels: horse, aurochs, wisent, cattle, neglect) aimed to decipher effects of different ungulate animals, or lack of them. We also ran CCAs for all possible nuisance covariables: year (both as 4-level factor and as a linear value), site (N vs. S), hour (two alternatives: a factor or 2nd-degree polynomial), weather (a combination of cloudiness and wind), nectar and plots position (forward-selected from latitude, longitude, their polynomials and interaction). We also tested for military vehicle effect (2-level factor tanks).

Next, in order to detect effects of Refaunation and Ungulates not attributable to nuisance effects of the covariates, we constructed two covariate models, one based on CANOCO forward selection from all the covariates that displayed significant effects in the single-term CCAs (FW model), the other based on manual backward elimination from all possible covariates (BW model). Linear combinations of all terms from FW and BW models were then entered as covariates to the refaunation and ungulates final models. These two final models were also explored for adding the effect of tanks.

Analogously to the past-present comparison, we interpreted the final CCA models (refaunation, refaunation + tanks, ungulates, ungulates + tanks) each controlled for FW and BW selected covariables, by the three sets of species’ attributes. We related the CCA scores to the three sets of attributes, using forward selection to identify the best-fitting attributes’ combinations.

## Supplementary Information

**Additional file 1.** Data on presence/absence of butterflies during three subsequent surveys of the (former) Milovice military training area, used for past-present comparison, together with life history traits, climatic niche traits, and conservation-related attributes of the species.

**Additional file 2.** Data on butterfly abundances *obtained *during current monitoring, plus environmental characteristics of monitoring plots, and life history, climatic niche, and conservation related-attributes of the species.

**Additional file 3.** CCA scores of individual butterfly species, plus species weights, from the *current monitoring* ordination analyses, final covariate models for refaunation + tanks and ungulates + tanks effects.

**Additional file 4.** Detailed results of the RDA analyses, interpreting the results of CCA analyses of refaunation / ungulates effects on butterfly assemblages by the constituent butterflies’ life history traits.

## Data Availability

All the primary data are included to this manuscript as electronic appendices.

## Abbreviations

BW: Backward-selected covariate model
CA: Correspondence analysis
CCA: Canonical correspondence analysis
FW: Forward-selected covariate model
RDA: Redundancy analysis
